# Anti-English Feeling in Greece

**Published:** 1912-12-21

**Authors:** Mariette Kephale

**Affiliations:** Secretary of Section for Hygiene and Nursing Union for Greek Women. Athens


### The Editor's Letter-Box.

### Anti-English Feeling in Greece.

*To the Editor of* The Hospital.

Sir,—I am a subscriber to your paper through a stationer in London and hasten to assure you that "Earnest" is incorrect in stating that there is an anti-English feeling prevailing in Greece to the extent of precluding Englishwomen from being employed as nurses. Their capacity for organising nursing staffs in our hospitals is a well-known fact and deeply appreciated.

If "Earnest" has heard from any partial and isolated case of this ill-disposed feeling against England it is to be attributed to the fact that there are Greeks who do not know the reason why, at the outset of this war, English public opinion and the English press have been so lacking in sympathy with this war, which is, above all, a just war to wipe out almost five centuries of wrong, cruelty, and every abomination the Turk is capable of, whereby he has made our Christian brethren suffer for so long.

Our cottage hospital has fifty beds, which we have arranged for wounded soldiers sent us by the First Military Hospital near by—in fact, an annexe; and it has been a source of great comfort to us that three English ladies, Mrs. Lloyd, Miss Acutt, and Mrs. Clifford-Culwick, and visitors to Athens have given their time and strength to help to nurse our men efficiently with their English practical way and devoted sense of duty that characterises your nation.

The Crown Princess also has many English nurses in her hospital. The English Red Cross Hospital also has done and is doing wonderful work in Salonica. Trusting that you will insert this in your valuable paper, believe me, sincerely yours,

Mariette Kephale,
Secretary of Section for Hygiene and Nursing Union for Greek Women.

Athens, December 2.

